# Semaglutide Reprograms Macrophages via the GLP-1R/PPARG/ACSL1 Pathway to Suppress Papillary Thyroid Carcinoma Growth

**DOI:** 10.1210/clinem/dgaf053

**Published:** 2025-02-05

**Authors:** Longlong Wang, Lele Zhang, Runsheng Ma, Yifei Zhang, Qungang Chang, Detao Yin

**Affiliations:** Department of Thyroid Surgery, the First Affiliated Hospital of Zhengzhou University, Engineering Research Center of Multidisciplinary Diagnosis and Treatment of Thyroid Cancer of Henan Province, Key Medicine Laboratory of Thyroid Cancer of Henan Province, Zhengzhou 450052, China; Department of Liver Transplantation, the First Affiliated Hospital of Zhengzhou University of Henan Province, Zhengzhou 450052, China; Department of Thyroid Surgery, the First Affiliated Hospital of Zhengzhou University, Engineering Research Center of Multidisciplinary Diagnosis and Treatment of Thyroid Cancer of Henan Province, Key Medicine Laboratory of Thyroid Cancer of Henan Province, Zhengzhou 450052, China; Department of Thyroid Surgery, the First Affiliated Hospital of Zhengzhou University, Engineering Research Center of Multidisciplinary Diagnosis and Treatment of Thyroid Cancer of Henan Province, Key Medicine Laboratory of Thyroid Cancer of Henan Province, Zhengzhou 450052, China; Department of Thyroid Surgery, the First Affiliated Hospital of Zhengzhou University, Engineering Research Center of Multidisciplinary Diagnosis and Treatment of Thyroid Cancer of Henan Province, Key Medicine Laboratory of Thyroid Cancer of Henan Province, Zhengzhou 450052, China; Department of Thyroid Surgery, the First Affiliated Hospital of Zhengzhou University, Engineering Research Center of Multidisciplinary Diagnosis and Treatment of Thyroid Cancer of Henan Province, Key Medicine Laboratory of Thyroid Cancer of Henan Province, Zhengzhou 450052, China

**Keywords:** semaglutide, tumor-associated macrophages, papillary thyroid carcinoma, GLP-1R signaling, macrophage polarization

## Abstract

**Context:**

The use of glucagon-like peptide-1 receptor (GLP-1R) agonists such as semaglutide, a novel class of antidiabetic medications, has raised concerns about potential adverse effects, particularly a possible association with thyroid cancer (TC).

**Objective:**

This study aims to evaluate whether semaglutide influences the progression of TC by modulating tumor-associated macrophages (TAMs).

**Methods:**

Semaglutide was administered to human papillary thyroid carcinoma (PTC) xenograft mouse models, coculture systems consisting of human THP-1 macrophage cells and PTC cells, and primary murine peritoneal macrophages. Assessments included tumor size, M1/M2 macrophage ratio, PTC cell proliferation, and polarization marker expression.

**Results:**

Semaglutide did not significantly impact the proliferation of PTC cells but reduced tumor size and inhibited the proliferation of PTC cells in coculture systems. It increased M1 and decreased M2 macrophages, reprogramming polarization by downregulating *PPARG* expression. Cotreatment with semaglutide and a *PPARG* agonist in the coculture system confirmed the upregulation of downstream genes *RSAD2*, *ACSL1*, and *PLA2G7*. Silencing *ACSL1* inhibited lipid accumulation in THP-1 cells and promoted polarization toward the M2 macrophage phenotype.

**Conclusion:**

Semaglutide modulates macrophage lipid metabolism through the GLP-1R/PPARG/ACSL1 signaling pathway. This modulation promotes the conversion of TAMs to the M1 macrophage phenotype, enhancing their anticancer activity. These findings suggest that semaglutide may improve therapeutic strategies, reduce unnecessary thyroid nodule screenings, and broaden its clinical applications.

Thyroid cancer (TC) is the most common malignant tumor of the endocrine system, with the majority (93%) originating from thyroid follicular cells and predominantly classified histologically as papillary thyroid carcinoma (PTC) (1). In contrast, medullary thyroid carcinoma (MTC), which arises from parafollicular C cells, is rare and accounts for only about 3% of TCs (2). The global incidence of TC is increasing rapidly, yet mortality rates have remained stable (3-5). Most new cases are papillary thyroid microcarcinomas, which are often detected in a subclinical state, grow slowly, and may cause few or no symptoms (4). Consequently, there is growing concern regarding the overdiagnosis and overtreatment of TC (3).

Glucagon-like peptide 1 (GLP-1) is an intestinally derived peptide secreted by L cells of the intestine, playing key roles in the physiological regulation of various organs, particularly in glucose and lipid metabolism (6, 7). GLP-1 receptor agonists (GLP-1RAs) have recently emerged as novel antidiabetic agents. By activating GLP-1 receptors (GLP-1Rs), these agonists stimulate insulin secretion in a glucose-dependent manner while inhibiting glucagon release (8). Semaglutide, a novel GLP-1RA with a prolonged half-life and enhanced stability against degradation by dipeptidyl peptidase-4, shows considerable promise in the treatment of metabolic diseases (9).

However, preclinical studies have suggested that GLP-1RAs might exert specific effects on the thyroid, potentially influencing TC development (10). Pharmacological studies in rodents suggest a potential link between GLP-1RAs and the development of MTC, which has led to black-box warnings for patients at risk of this condition (10, 11). However, GLP-1R expression is rarely found in human C-cell tumors, and the carcinogenicity observed in animal studies has not been definitively confirmed in humans. A case-control study involving 47 746 patients with diabetes suggested an association between GLP-1RAs use and increased risks of both PTC and MTC (12). The study recommended heightened monitoring of TC risk factors in patients initiating GLP-1RA therapy. However, another cohort study investigating the risk of 13 obesity-related cancers in patients with type 2 diabetes indicated that there is no statistically significant difference in the risk of thyroid cancer between patients treated with GLP-1RAs and those treated with insulin (13). While research on the relationship between GLP-1RAs and TC has increased, conclusive evidence remains elusive (14). Excessive concern over unsubstantiated TC risks could lead to underutilization of GLP-1RAs in patients who could greatly benefit from them, as well as unnecessary screening and overdiagnosis of TC.

The tumor microenvironment (TME) comprises various cellular and noncellular components, with inflammation recognized as a significant factor influencing cancer progression (15). Among the immune cells infiltrating the TME, tumor-associated macrophages (TAMs) play a pivotal role. Previous studies suggest that GLP-1RAs may modulate macrophage polarization at various sites, leading to either beneficial or pathological effects (16-18). Although some studies (19, 20) have investigated the impact of GLP-1RAs on energy metabolism in human PTC cells, it remains unclear whether these drugs can influence TC progression by affecting the inflammatory microenvironment or acting on immune cells. This study aims to explore the effects of semaglutide on TAM polarization and its potential role in the progression of PTC. Our findings reveal that semaglutide does not directly affect PTC cells but modulates *PPARG* expression in TAMs, inhibiting their polarization toward the M2 phenotype and, in turn, suppressing the proliferation of TC cells.

## Methods

### Patient Tissues

Thyroid cancer and adjacent tissue specimens used in this study were collected from the Department of Thyroid Surgery at the First Affiliated Hospital of Zhengzhou University, Zhengzhou, China, between 2021 and 2024. All specimens were primary and had not undergone any treatment prior to surgical intervention. Informed written consent was obtained from all participants. The use of human tissues complied with the principles outlined in the Declaration of Helsinki and received approval from the Institutional Ethics Committee of the First Affiliated Hospital of Zhengzhou University (Approval No. 2020-KY-0075-002).

### Key Resource and Cell Culture

The key reagents, resources, and cell lines used in this study are listed in Supplementary Table S1 (21). All cell lines underwent authentication and mycoplasma testing. Cells were cultured in either high-glucose DMEM or RPMI 1640 medium, supplemented with 10% fetal bovine serum (FBS) and 1% penicillin/streptomycin, in a humidified 5% CO_2_ atmosphere at 37 °C.

### Mice

Male BALB/c nude and C57BL/6 mice (5 weeks old) were housed in a specific-pathogen-free environment, with unrestricted access to water and standard chow. They were maintained on a 12-hour light-dark cycle at a temperature of 24 ± 2 °C and a relative humidity of 40% to 60%. All procedures involving mice were approved by the Institutional Life Science Ethics Committee of Zhengzhou University (Approval No. ZZU-LAC20231222 [04]).

### Cell Viability Assay

For the CCK-8 assay, cells were seeded in 96-well plates at a density of 2 × 10^3^ cells per well, with 3 replicates per sample. After a 24-hour adhesion period, the medium was replaced with fresh medium containing different concentrations of semaglutide (0nM, 10nM, 100nM, 1000nM). At various time points (24, 48, and 72 hours), 10 μL of CCK-8 solution (Dojindo) was added to each well and incubated at 37 °C in a humidified 5% CO_2_ environment for 1 to 2 hours. Absorbance at 450 nm was then measured for each well.

### EdU Assay

For the EdU assay, cells were seeded into 24-well plates at a density of 2 × 10^4^ cells per well, with 3 replicates per sample. After a 24-hour adhesion period, the medium was replaced with fresh medium containing different concentrations of semaglutide (0nM, 10nM, 100nM, 1000nM). Following a 48-hour incubation, EdU working solution (Beyotime) was added to each well and incubated at 37 °C for 1 to 2 hours. Cells were then fixed with 4% paraformaldehyde for 15 minutes and permeabilized with 0.3% Triton X-100 in phosphate-buffered saline (PBS) for 15 minutes. EdU staining was performed, followed by Hoechst staining to visualize the nuclei. The results were observed under a fluorescence microscope.

### Quantitative Real-Time Polymerase Chain Reaction

For quantitative real-time polymerase chain reaction (qPCR), total RNA was extracted using TRIzol™ reagent (Invitrogen) following the manufacturer's instructions. RNA purity was assessed with a NanoDrop One spectrophotometer (Thermo Fisher). The RNA was then reverse-transcribed into cDNA using the PrimeScript™ RT reagent kit (Takara). The qPCR reactions were performed in a total volume of 20 μL using Tli RNaseH Plus (Takara) on a 7300 Fast Real-Time PCR System (Thermo Fisher). mRNA expression levels were normalized to *GAPDH* and calculated using the 2^−ΔΔCt^ method. Primer sequences are available in Supplementary Table S2 (21).

### Western Blot

Proteins from tissues and cells were separated on 8% to 12% SDS-PAGE (sodium dodecyl sulfate–polyacrylamide gel electrophoresis) gels and transferred onto PVDF membranes (Merck). The membranes were blocked with 5% bovine serum albumin and incubated overnight at 4 °C with primary antibodies. The next day, the membranes were incubated with secondary antibodies for 1 hour at room temperature. After 3 washes with Tris-buffered saline containing 0.1% Tween 20, the blots were developed using an enhanced chemiluminescence substrate (GenView) and imaged using an Amersham Imager 680 (GE Health). The following antibodies were used: rabbit anti-GLP-1R (Catalog # NBP1-97308; RRID: AB_11139100), rabbit anti-GAPDH (Catalog # 10494-1-AP; RRID: AB_2263076) and goat anti-rabbit IgG (Catalog # 31460; RRID: AB_228341) (21).

### In Vivo Antitumor Therapy

To assess the antitumor effects of semaglutide in vivo, a total of 2 × 10^6^ IHH-4 or TPC-1 cells were subcutaneously injected into the right axilla of the BALB/c mice. Once the tumors became palpable, the mice were randomly divided into 4 groups (n = 6 per group) and received subcutaneous injections of PBS containing different concentrations of semaglutide (0 μg/kg·d, 10 μg/kg·d, 60 μg/kg·d, and 120 μg/kg·d). Body weight, fasting blood glucose, and tumor volume (calculated as 1/2 × length × width^2^) were monitored every 3 days from the first dose. When tumors in any group reached approximately 2 × 10^3^ mm^3^, the mice were euthanized. Tumors were excised for measurement and histological analysis, and blood samples were collected for biochemical analysis.

### Flow Cytometry

For macrophage polarization analysis, tumors from mice or patients were homogenized in Hanks' balanced salt solution containing 1 mg/mL collagenase type II (Sigma-Aldrich), 0.1 mg/mL hyaluronidase (Solarbio), 0.1 mg/mL DNase I (Roche), 5% FBS, and 10 mM HEPES (Beyotime) and digested for 1 hour at 37 °C under gentle agitation. The homogenate was filtered to obtain a single-cell suspension. Tumor-infiltrating immune cells were isolated by centrifugation using 40% and 80% Percoll solutions. These immune cells were incubated with fluorescence-conjugated antibodies for 30 minutes at 4 °C. Then the cells were detected using BD FACSCelesta (BD Biosciences) and data were analyzed using FlowJo software (USA). The following antibodies were used: FITC anti-mouse CD45 (Catalog # 147709; RRID: AB_2563541), Brilliant Violet 510 anti-mouse CD11b (Catalog # 101245; RRID: AB_2561390), PE anti-mouse F4/80 (Catalog # 123109; RRID: AB_2563543), PE/Cyanine7 anti-mouse CD86 (Catalog # 105013; RRID: AB_439782), APC anti-mouse CD206 (Catalog # 141707; RRID: AB_10896057), FITC anti-human CD45 (Catalog # 304005; RRID: AB_314393), Brilliant Violet 421 anti-human CD11b (Catalog # 301323; RRID: AB_10933087), APC anti-human CD80 (Catalog # 305219; RRID: AB_2291403), PE anti-human CD206 (Catalog # 321105; RRID: AB_571910).

### Histology and Immunohistochemistry

Tumor samples from mice were fixed in 4% paraformaldehyde overnight with gentle agitation. The samples were dehydrated in 75% ethanol, embedded in paraffin, and sectioned into 4- to 6-μm slices. Paraffin sections were dewaxed, rehydrated, and incubated in 3% H₂O₂ in methanol for 10 minutes to block endogenous peroxidase activity. For hematoxylin and eosin (H&E) staining, slides were stained with hematoxylin-eosin and imaged using an Eclipse Ni-U microscope (Nikon). For immunohistochemistry, heat-induced antigen retrieval was performed using a 10 mmol/L pH 6.0 solution. Slides were blocked in 5% goat serum for 1 hour and then incubated overnight at 4 °C with primary antibodies. The following day, the slides were washed with PBS and incubated with secondary antibodies at 37 °C for 1 hour. Visualization was achieved with diaminobenzidine solution, followed by light counterstaining with hematoxylin. Sections were imaged using an Eclipse Ni-U microscope (Nikon). The following primary antibodies were used: rabbit anti-Ki67 (Catalog # ab15580; RRID: AB_443209) and rabbit anti-PCNA (Catalog # ab92552; RRID: AB_10561973) (21).

### In Vitro Coculture and siRNA Transfection

THP-1 monocytes were seeded into Transwell inserts or microwells and treated with 100nM phorbol myristate acetate for 24 hours to induce differentiation into macrophages. Simultaneously, TPC-1 or IHH4 cells were plated in microwells or Transwell inserts and allowed to adhere for 24 hours. After this, the Transwell inserts were placed onto the microwells, and fresh medium containing 100nM semaglutide was added. The cultures were then incubated for an additional 48 hours before harvesting the cells from the microwells.

For RNA interference experiments, THP-1 cells were seeded into microwells and treated with 100nM phorbol myristate acetate for 24 hours to induce differentiation. Transfection was performed using siRNA targeting *ACSL1* (sequence: 5′-GTGGGTGATTATTGAACAA-3′) and Lipofectamine 3000. Final concentrations were 20nM for siRNA and 1.6 µg/mL for Lipofectamine 3000. After 48 hours of transfection, the cells underwent the same coculture conditions as previously described.

### Isolation and Culture of Primary Mouse Peritoneal Macrophages

C57BL/6 mice were intraperitoneally injected with 3% thioglycollate medium (Brewer) for 3 consecutive days and euthanized on the fourth day. After euthanasia, the peritoneal cavity was flushed with PBS to collect peritoneal cells. The collected cells were centrifuged, and the pellet was resuspended in ACK lysis buffer for 3 minutes to lyse red blood cells. The remaining cells were cultured in RPMI-1640 medium supplemented with 10% FBS at 37 °C for 4 hours. Nonadherent cells were removed by washing with PBS, and the adherent cells were identified as peritoneal macrophages (PMs). To induce M1 or M2 polarization, PMs were incubated in RPMI-1640 medium containing 10% FBS with 100 ng/mL lipopolysaccharide (LPS; Solarbio) or 20 ng/mL interleukin-4 (IL-4; PeproTech) for 48 hours. To assess the effect of semaglutide, PMs were pretreated with RPMI-1640 medium, with or without 100nM semaglutide, for 6 hours prior to polarization.

### RNA-Seq

Total RNA was extracted using the TRIzol method, and its concentration and integrity were assessed. Library construction and RNA sequencing (RNA-Seq) were performed by Meiwei Metabolic Biotechnology Co., Ltd. (Wuhan, China) using the Illumina NovaSeq 6000 platform (Illumina). Differential gene expression analysis between groups was conducted using DESeq2, with significance thresholds based on false discovery rate (FDR) and log_2_ fold change. Differentially expressed genes (DEGs) were identified using the criteria of |log_2_ Fold Change| ≥ 2 and *FDR* < 0.01.

### Oil Red O Staining

THP-1 cells from the coculture system were collected and fixed with 4% paraformaldehyde. Fresh Oil Red O staining solution (Solarbio) was prepared according to the manufacturer's instructions and used to stain the cells at room temperature, protected from light, for 30 minutes. The cells were then washed with 60% isopropanol and counterstained with Mayer's hematoxylin to visualize the nuclei. Staining results were observed under a microscope. To quantify the staining, 100% isopropanol was added to dissolve the Oil Red O stain, and absorbance at 500 nm was measured for each well.

### Statistical Analysis

Statistical analyses were conducted using GraphPad Prism 9.5 software (USA). Quantitative data were presented as mean ± SD. Statistical differences were assessed using either a two-tailed unpaired Student *t* test or one-way ANOVA, as appropriate. Pearson correlation values and associated *P* values were calculated between gene expressions and proportion of macrophages. In all figures, significance levels are indicated as follows: * *P* < .05, ** *P* < .01, and *** *P* < .001. Differences were considered statistically significant at *P* < .05.

## Results

### Semaglutide Fails to Elicit Significant Effects on the Proliferation of PTC Cells

**Figure 1.**
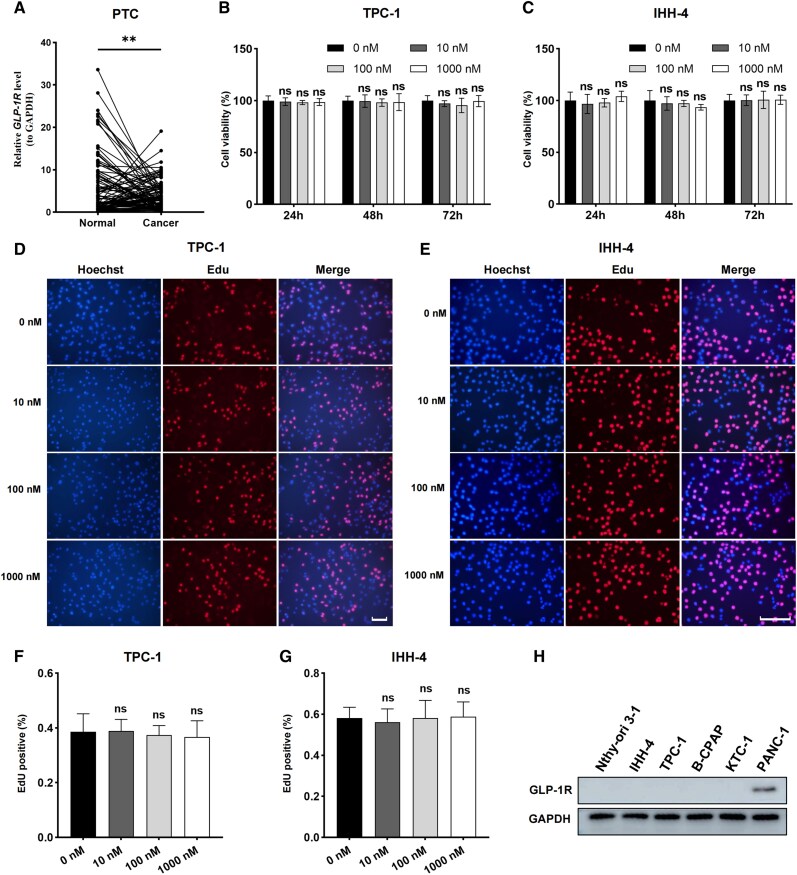
Semaglutide's limited impact on PTC cell proliferation. (A) GLP-1R mRNA expression was measured in 96 pairs of PTC tumor and adjacent normal tissue samples via qPCR. (B, C) The proliferation of TPC-1 and IHH-4 cells was evaluated over 24, 48, and 72 hours using the CCK-8 kit at semaglutide concentrations of 0nM, 10nM, 100nM, and 1000nM. (D-G) DNA replication activity in TPC-1 (D, F) and IHH-4 (E, G) cells was assessed using the EdU assay after 48 hours of treatment with semaglutide at concentrations of 0nM, 10nM, 100nM, and 1000nM. Scale bar: 100 μm. (H) GLP-1R protein levels were examined in normal thyroid follicular cell line (Nthy-ori 3-1), human PTC cell lines (IHH-4, TPC-1, B-CPAP, KTC-1), and positive control human prostate cancer cell line (PANC-1) by Western blotting. Data are presented as mean ± SD. *P < .05, **P < .01, ***P < .001.

Abbreviation: PTC, papillary thyroid carcinoma.

To assess the potential effects of semaglutide on PTC cells, we first examined the expression of GLP-1R in 96 pairs of PTC tumor and adjacent normal tissue samples (Fig. 1A). Our analysis revealed a decreased expression of GLP-1R in PTC tissues. We assessed the correlation of GLP-1R expression with clinical pathology factors. As shown in Table 1, there was a significant association between GLP-1R expression and tumor size. Next, we evaluated the impact of semaglutide on the viability of PTC cells using TPC-1 and IHH-4 cell lines (Fig. 1B-1G). CCK-8 assays showed that semaglutide, at concentrations ranging from 10nM to 1000nM over 24 to 72 hours, did not significantly affect the proliferation of TPC-1 or IHH-4 cells (Fig. 1B and 1C). Likewise, EdU assays demonstrated no statistically significant differences in DNA replication activity in these cells after treatment with semaglutide at 10nM to 1000nM for 48 hours (Fig. 1D-1G). Further analysis of GLP-1R protein expression across normal thyroid follicular cell line and several PTC cell lines revealed minimal or absent expression (Fig. 1H) (22). Given that semaglutide functions as a long-acting GLP-1R agonist, its lack of effect on PTC cell proliferation could be explained by the low GLP-1R expression in these cells. In the TME, there may be other GLP-1R-expressing cells, like immune cells and fibroblasts, crucial in tumor growth, angiogenesis, and immune responses. Further studies are needed to explore whether semaglutide might affect tumor progression through interactions with other cell types within the TME.

**Table 1.** Correlation between GLP-1R expression and clinicopathological features in 96 papillary thyroid carcinoma patients

Clinicopathological features	Group	No. of patients, *N* = 96 (%)	*P* value
Age, years	< 55	74 (77.1)	*P* = .322
	≥ 55	22 (22.9)	
Tumor size, cm	< 2	54 (56.3)	*P* = .013
	≥ 2	42 (43.7)	
Extrathyroidal extension	No	76 (79.2)	*P* = .455
	Yes	20 (20.8)	
Lymph node metastasis	No	62 (64.6)	*P* = .563
	Yes	34 (35.4)	

Differences between experimental groups were analyzed by Mann-Whitney test.

### Semaglutide Inhibits Thyroid Cancer Cell Proliferation and Tumor Progression in Xenograft Mouse Models

**Figure 2.**
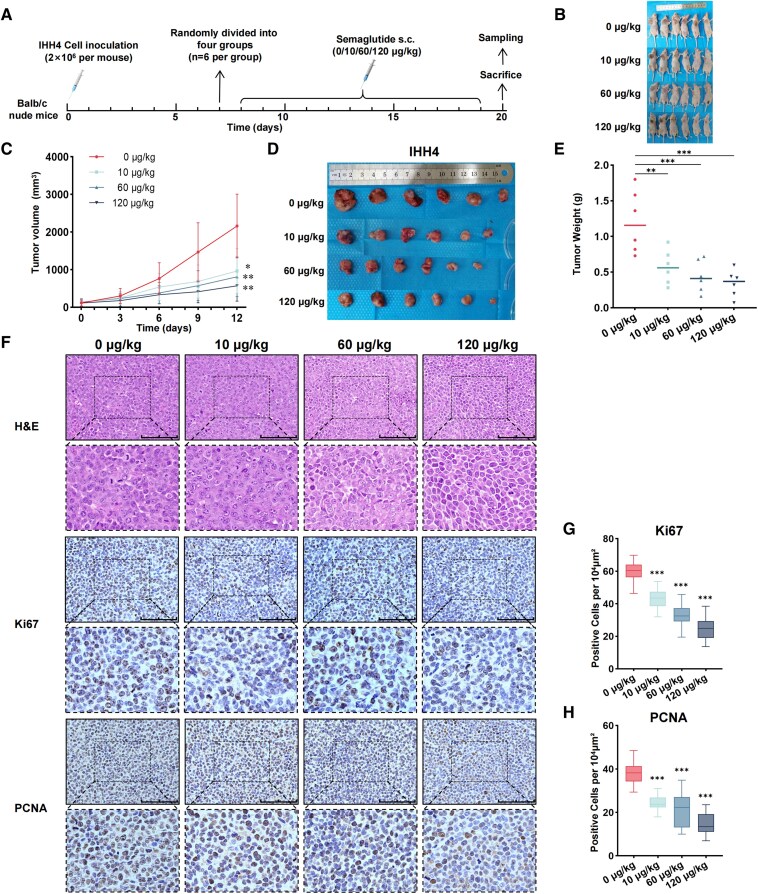
Semaglutide inhibits IHH4 cell proliferation and tumor progression in vivo (n = 6 per group). (A) Experimental design: Balb/c nude mice were implanted with IHH4 cells and treated daily with different doses of semaglutide via subcutaneous injections. The mice were sacrificed after 12 days of treatment. (B) Whole-body image of a mouse postmortem. (C) Tumor growth curves. (D) Tumor volume. (E) Tumor weight. (F-H) Pathological and cellular proliferation changes in xenograft tumors were assessed by H&E staining, Ki67 staining, and PCNA staining. The density of Ki67+ and PCNA+ tumor cells was calculated. Scale bar: 100 μm. Results are shown as mean ± SD. *P < .05, **P < .01, ***P < .001.

To investigate whether semaglutide affects tumor progression through the TME, we conducted in vivo experiments using a human IHH-4 cell xenograft model in Balb/c nude mice (Fig. 2A).

Our findings indicated that semaglutide significantly suppressed the rate of tumor growth (Fig. 2B and 2C). While no statistically significant differences were observed among the treatment groups, the tumor volume change curve suggested a dose-dependent inhibition trend. Dissection and weighing of the tumors further confirmed these findings, showing significantly reduced tumor weights in the semaglutide-treated groups compared to the control group (Fig. 2D and 2E). Immunohistochemistry analysis also demonstrated significantly decreased levels of ki67 and proliferating cell nuclear antigen (PCNA) expression in the treated groups, indicating reduced cellular proliferation rates (Fig. 2F-2H).

Throughout the experiment, we monitored changes in mouse body weight and blood glucose levels. Semaglutide treatment led to a reduction in body weight, with the effect becoming more pronounced at higher drug concentrations (Supplementary Fig. S1A) (21). However, semaglutide did not significantly impact blood glucose levels (Supplementary Fig. S1B) (21). After euthanasia, we analyzed liver and kidney function, cardiac enzymes, and other biochemical parameters (Supplementary Fig. S1C) (21). Administration of semaglutide at various concentrations did not significantly alter these biochemical levels compared to the control group. These results suggest that semaglutide is safe within the tested dose range in this mouse model.

### Semaglutide Promotes M2 to M1 Macrophage Polarization in the TME

The immune system plays a crucial role in shaping the TME. Macrophages, key components of innate immunity, exhibit various polarization states that can significantly influence tumor progression (23). We investigated whether semaglutide could impact tumor progression by altering the polarization state of TAMs.

**Figure 3.**
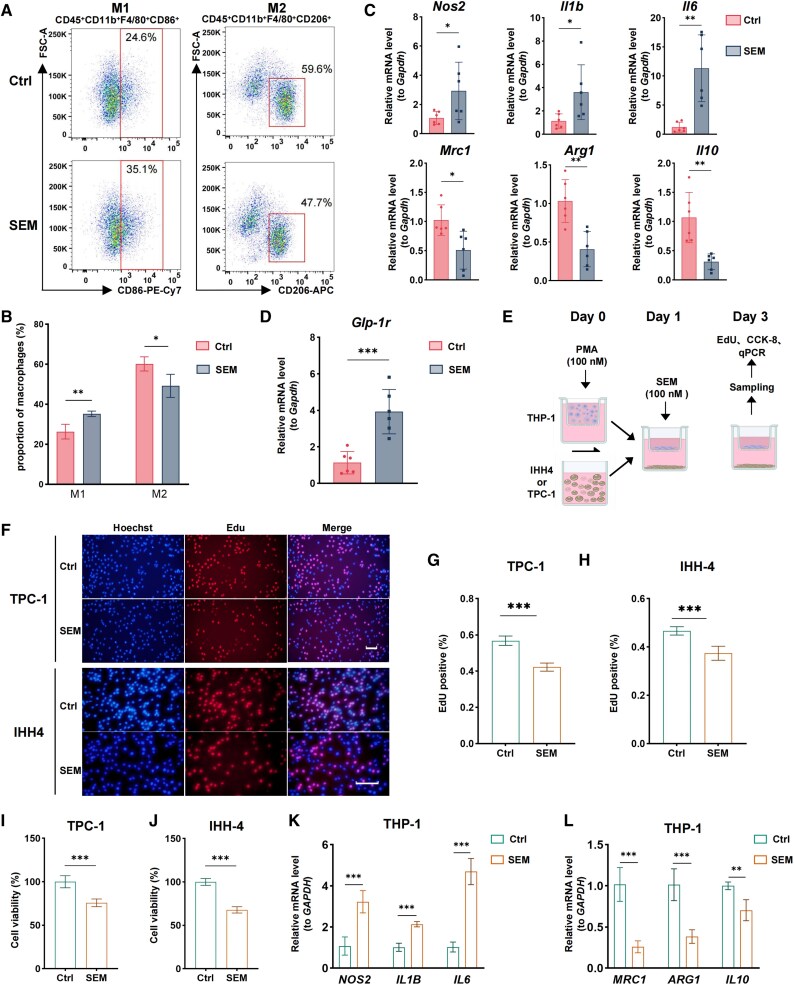
Semaglutide's role in modulating macrophage polarization and PTC suppression. (A, B) Flow cytometry was employed to analyze the proportions of M1 and M2 macrophages in TPC-1 xenograft tumors derived from mice subjected to subcutaneous injections of 120 μg/kg semaglutide (SEM) or PBS (Ctrl) for 2 consecutive weeks. (C) qPCR was utilized to measure the relative mRNA levels of M1 macrophage markers (*Nos2, Il1b, Il6*) and M2 markers (*Mrc1, Arg1, Il10*) in the aforementioned xenograft tumors. (D) qPCR was utilized to measure the relative mRNA levels of Glp-1r in the aforementioned xenograft tumors. (E) Experimental outline for in vitro coculture experiments. THP-1 and IHH-4 (or TPC-1) cells were seeded into Transwell inserts or microwells and allowed to adhere separately. Then, the cells were cocultured for 48 hours in medium with (SEM) or without (Ctrl) 100nM semaglutide. At last, tumor cells were harvested and evaluated for cell viability using CCK-8 or EdU assays, and THP-1 cells were collected and analyzed for the expression of M1 and M2 marker genes by qPCR. (F-H) The EdU assay was used to evaluate DNA replication activity in TPC-1 and IHH-4 cells treated with semaglutide in a coculture system for 48 hours. Scale bar: 100 μm. (I-J) The CCK-8 kit was used to measure the proliferation of TPC-1 and IHH-4 cells in coculture after semaglutide treatment. (K-L) Relative mRNA levels of M1 (K) and M2 (L) macrophage markers in THP-1 cells within the coculture system were analyzed by qPCR. Data are presented as mean ± SD. *P < .05, **P < .01, ***P < .001.

First, we established a xenograft model using human TPC-1 cells in nude mice. The treatment group (SEM) received subcutaneous injections of 120 μg/kg semaglutide, while the control group (Ctrl) received PBS. Consistently with previous results, semaglutide significantly inhibited TPC-1 cell proliferation in vivo (Supplementary Fig. S2A-S2D) (21). Immune phenotyping was performed on the immune cells extracted from the tumor tissues using flow cytometry (Supplementary Fig. S2E) (21). Compared to the control group, the treatment group exhibited a notable increase in M1-like TAMs (CD45^+^CD11b^+^F4/80^+^CD86^+^) and a decrease in M2-like TAMs (CD45^+^CD11b^+^F4/80^+^CD206^+^) (Fig. 3A and 3B). The qPCR analysis of total mRNA from tumor tissues revealed significant upregulation of M1 markers *Nos2*, *Il1b*, and *Il6*, along with marked downregulation of M2 markers *Mcr1*, *Arg1*, and *Il10* (Fig. 3C). Additionally, the mRNA level of *Glp-1r* was significantly increased in the treatment group (Fig. 3D). These results implied that semaglutide may promote M2 to M1 macrophage polarization in the TME by activating GLP-1R.

Next, we cocultured human THP-1 cells with TPC-1 or IHH-4 thyroid cancer cells. The cocultured cells were divided into treatment (SEM) and control (Ctrl) groups, receiving 100nM semaglutide or control medium for 48 hours (Fig. 3E). Tumor cells TPC-1 or IHH-4 were then harvested and evaluated for cell viability. EdU staining indicated that DNA replication activity was significantly lower in the semaglutide-treated group (Fig. 3F-3H). Similarly, CCK-8 assays showed a substantial reduction in cell proliferation in the treatment group compared to the control group (Fig. 3I and 3J). Additionally, total mRNA from THP-1 cells in the coculture system was analyzed by qPCR, revealing significant upregulation of M1 markers *NOS2*, *IL1B*, and *IL6*, and downregulation of M2 markers *MCR1*, *ARG1*, and *IL10* in TAMs (Fig. 3K, 3L). These findings indicate that semaglutide inhibits tumor progression by reprogramming TAMs, shifting them from an M2 to an M1 polarization state.

### Semaglutide Modulates Macrophage Polarization by Suppressing PPARG Expression

**Figure 4.**
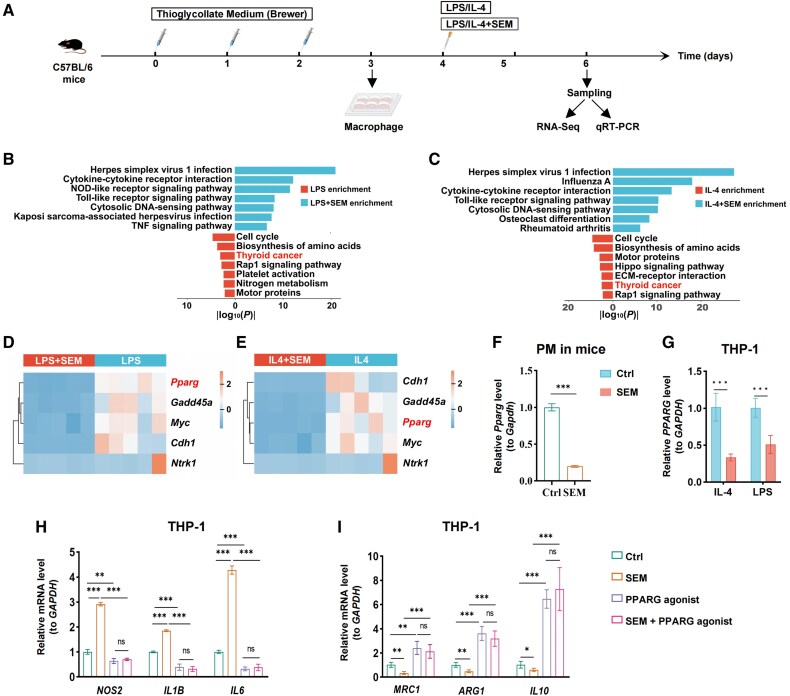
Semaglutide modulates macrophage polarization via PPARG suppression. (A) Experimental design: C57BL/6 mice were intraperitoneally injected with Brewer thioglycollate medium for 3 days. Peritoneal macrophages (PMs) were then collected and cultured for 6 hours with or without 100nM semaglutide, followed by polarization with LPS or IL-4 for 48 hours. Total mRNA was analyzed by RNA-Seq and qPCR. (B, C) Bar chart visualizes the KEGG enrichment analysis for downregulated genes from RNA-Seq data across various treatment and control groups. LPS: LPS Ctrl group; LPS + SEM: LPS + semaglutide–treated group; IL-4: IL-4 Ctrl group; IL-4 + SEM: IL-4 + semaglutide–treated groups. Blue, upregulated in PMs treated with semaglutide; Red, downregulated in PMs treated with semaglutide. (D, E) A heatmap displays the genes enriched in the Thyroid cancer pathway. (F) qPCR analysis of relative *Pparg* expression levels in PMs across semaglutide treatment (SEM) and control (Ctrl) groups. (G) qPCR analysis of the relative expression levels of *PPARG* in THP-1 cells in the coculture system from the previous part. (H) The effects semaglutide and a PPARG agonist on the expression of M1 (*NOS2, IL1B, IL6*) and M2 (*MRC1, ARG1, IL10*) macrophage markers in THP-1 cells were assessed by qPCR. Data are presented as mean ± SD.

Abbreviations: Ctrl, control; SEM, semaglutide; PM, peritoneal macrophage. *P < .05, **P < .01, ***P < .001.

To elucidate the molecular mechanism by which semaglutide regulates TAM polarization, we conducted a series of in vitro experiments using primary PMs isolated from C57BL/6 mice. First, the collected PMs were cultured in a complete medium with or without 100nM semaglutide for 6 hours, followed by polarization induction with either LPS or IL-4 for 48 hours. After the treatments, total mRNA was extracted and analyzed using RNA-Seq and qPCR (Fig. 4A).

Principal component analysis of RNA-Seq data revealed significant differences in gene expression between the semaglutide-treated groups and controls (Supplementary Fig. S3A and S3B) (21). Volcano plots identified DEGs between the semaglutide-treated groups and controls (IL-4 + SEM vs IL-4, LPS + SEM vs LPS) (Supplementary Fig. S3C and S3D) (21). A heatmap of established macrophage polarization markers was also generated (Supplementary Fig. S3E and S3F) (21, 24-27). The data indicated that M1 macrophage markers were upregulated, while M2 markers were downregulated in the semaglutide-treated group, consistent with our prior coculture findings. Subsequently, we performed enrichment analysis on DEGs (Fig. 4B and 4C and Supplementary Fig. S3G) (21). The KEGG enrichment analysis revealed that the Thyroid cancer signaling pathway was significantly enriched in the downregulated genes of both the IL-4 and LPS treatment groups (Fig. 4B and 4C). A heatmap displayed the enrichment of 5 genes in this pathway, among which PPARG exhibited the most significant inhibition (Fig. 4D and 4E).

To confirm whether reduced *PPARG* expression is a key factor in semaglutide-mediated inhibition of M2 macrophage polarization, we first measured *PPARG* mRNA levels. The qPCR results showed that semaglutide suppressed *PPARG* expression in mouse PMs treated with IL-4 or LPS (Fig. 4F), as well as in THP-1 cells within the coculture system (Fig. 4G). In the THP-1 and TPC-1/IHH-4 coculture system, the cells were first pretreated with Pparg agonist for 6 hours, followed by coculture for 48 hours in medium with or without 100nM semaglutide. The THP-1 cells were then collected. The qPCR results demonstrated that this treatment significantly attenuated semaglutide-induced upregulation of M1 macrophage markers (*NOS2*, *IL1B*, and *IL6*), and mitigated the downregulation of M2 macrophage markers (*MCR1*, *ARG1*, and *IL10*) (Fig. 4H). Notably, cotreatment with the *PPARG* agonist and semaglutide resulted in no significant difference in M1 and M2 marker expression compared to treatment with the agonist alone (Fig. 4H). These findings suggest that PPARG plays a pivotal role as a downstream regulator in semaglutide's modulation of TAM polarization.

### PPARG Reprograms Macrophage Polarization by Regulating the Expression of Downstream Genes

**Figure 5.**
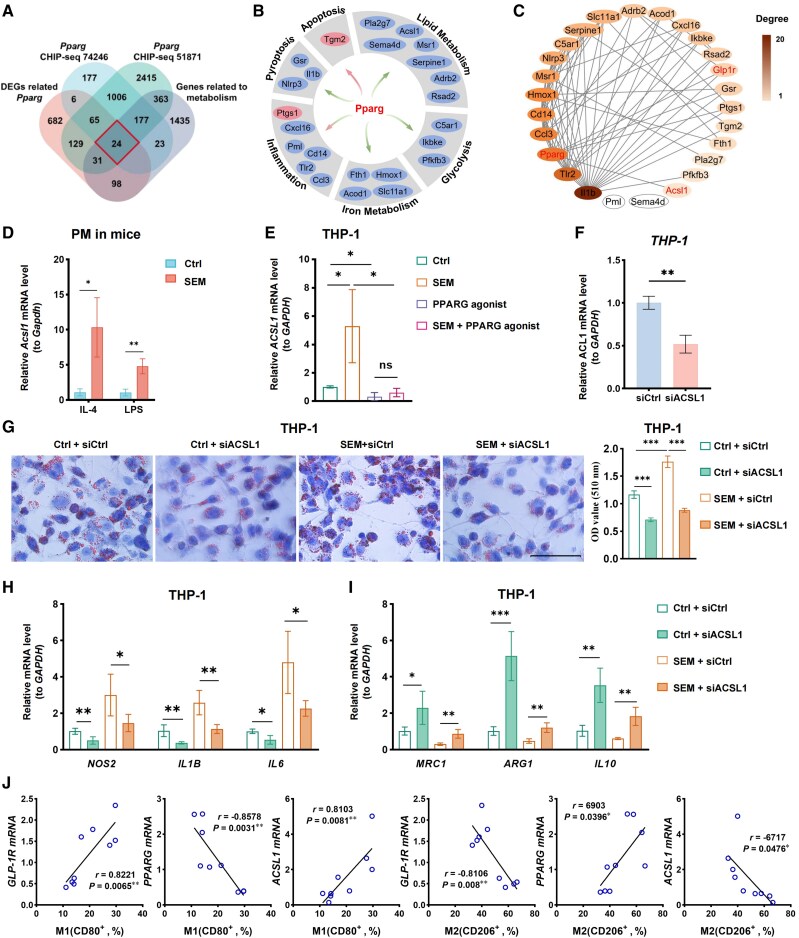
Macrophage metabolism and polarization regulated by the PPARG-ACSL1 axis. (A) Venn diagram showing the overlap of PPARG-associated differentially expressed genes (from ChIP-seq data in the Cistrome Data Browser) with metabolism-related genes from the NCBI Gene database. (B) Pie chart depicting the classification and regulatory pattern of potential downstream genes of PPARG. Red circles indicate positive regulation, and blue circles indicate negative regulation. (C) The protein-protein interaction (PPI) network for GLP-1R, PPARG, and their downstream target genes was generated using the STRING database. (D-E) ACSL1 expression levels were measured in primary peritoneal macrophages from mice (D) and in THP-1 cells in a coculture system (E). (F) ACSL1 mRNA expression was measured in THP-1 cells treated with either siCtrl or siACSL1 in a coculture system via qPCR. (G) Lipid accumulation in THP-1 cells within the coculture system was evaluated using Oil Red O staining. (H-I) Relative expression of M1 markers (*NOS2*, *IL1B*, *IL6*) and M2 macrophage marker (*MRC1, ARG1, IL10*) in THP-1 cells within the coculture system was analyzed by qPCR. (J) The proportion of M1 macrophages (CD45^+^CD11b^+^CD80^+^) or M2 macrophages (CD45^+^CD11b^+^CD206^+^) in papillary thyroid carcinoma from patients were plotted against the relative mRNA levels of *GLP-1R*, *PPARG* and *ACSL1* (n = 9). Linear regression analysis was performed for each scatterplot. Data are presented as mean ± SD.

Abbreviation: PM, peritoneal macrophage. *P < .05, **P < .01, ***P < .001.

*PPARG* encodes a transcription factor that plays critical roles in lipid metabolism, glucose metabolism, and inflammation (28, 29). To explore its involvement in TAM polarization, we first identified DEGs related to *PPARG* expression through Pearson correlation analysis. We then used ChIP-seq data from the Cistrome Data Browser (Data IDs 74246 and 51871) to identify PPARG binding sites in mouse macrophages. Additionally, we retrieved a list of metabolism-related genes from the NCBI gene database. By intersecting these datasets, we identified 24 potential downstream target genes (Fig. 5A and 5B). We further used the STRING database to predict the protein-protein interaction network involving PPARG and its downstream molecules, regulated by semaglutide via GLP-1R (Fig. 5C).

Since macrophage activation is closely linked to its metabolic state, we analyzed the expression of genes involved in lipid and glucose metabolism using qPCR (Supplementary Fig. S4 (21), Fig. 5D). Our results showed that semaglutide treatment increased the expression of *RSAD2*, *ACSL1*, *PLA2G7*, *SERPINE1*, *PFKB3, C5AR1*and *IKBKE* in IL-4 or LPS-stimulated mouse peritoneal macrophages (PMs) (Supplementary Fig. S4) (21) and in THP-1 cells within coculture systems (Fig. 5D). However, when a PPARG agonist was introduced into the coculture system, the upregulation of *RSAD2*, *ACSL1*, and *PLA2G7* gene expression was suppressed (Fig. 5D). Due to the protein-protein interaction (PPI) analysis revealing potential interactions between RSAD2 and PLA2G7 with other DEGs (Fig. 5C), we subsequently concentrated on the ACSL1 gene, which is uniquely associated with PPARG in the PPI analysis.

To further assess the role of *ACSL1* in TAM polarization, we silenced *ACSL1* expression using siRNA in THP-1 cells in a coculture system. The qPCR analysis confirmed that the siACSL1 group showed significantly reduced *ACSL1* expression compared to the siCtrl group (Fig. 5F). Given its role in lipid and fatty acid metabolism (30), particularly in the mitochondria and endoplasmic reticulum, we examined the connection between macrophage lipid metabolism and polarization. Oil Red O staining revealed that *ACSL1* silencing significantly reduced lipid droplet accumulation in THP-1 cells treated with somatropin (Fig. 5G).

We then analyzed the impact of *ACSL1* silencing on macrophage polarization. The qPCR results demonstrated that silencing *ACSL1* significantly decreased the expression of M1 macrophage markers (Fig. 5H), while increasing the expression of M2 macrophage markers (Fig. 5I). These findings suggest that *ACSL1* gene silencing promotes M2 macrophage polarization.

**Figure 6.**
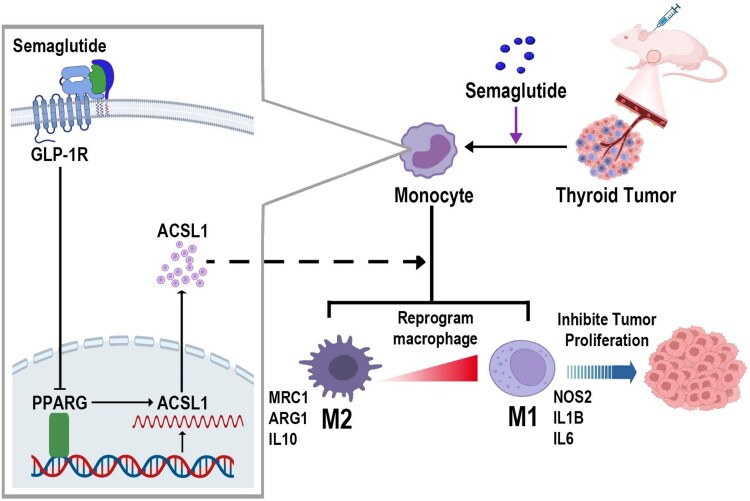
Schematic representation of semaglutide's impact on thyroid cancer proliferation and molecular mechanisms. semaglutide modulates macrophage polarization via the GLP-1R/PPARG/ACSL1 signaling pathway, leading to the induction of M1 polarization in TAMs and contributing to its antitumor effects.

Our findings were validated using xenograft tumor tissues from previous experiments and clinical samples from patients with PTC (Supplementary Fig. S5 (21), Fig. 5J). The qPCR analysis of xenograft tumors from mice showed that *Glp-1r* mRNA expression was negatively associated with *Pparg* mRNA expression and positively correlated with *Acsl1* mRNA expression (Supplementary Fig. S5A) (21). Flow cytometry and qPCR analysis of clinical samples revealed that the proportion of M1 macrophages (CD45^+^CD11b^+^CD80^+^) was positively associated with *GLP-1R* and *ACSL1* mRNA expression and negatively correlated with *PPARG* mRNA expression (Supplementary Fig. S5B (21), Fig. 5J). Conversely, the proportion of M2 macrophages (CD45^+^CD11b^+^CD206^+^) was positively associated with *PPARG* mRNA expression and negatively correlated with *GLP-1R* and *ACSL1* mRNA expression. In conclusion, semaglutide modulates macrophage lipid metabolism and polarization via the GLP-1R/PPARG/ACSL1 signaling pathway, leading to the induction of M1 polarization in TAMs and contributing to its antitumor effects (Fig. 6).

## Discussion

Various metabolic disorders are intricately associated with thyroid diseases (31). Epidemiological research has demonstrated that obesity and diabetes frequently serve as prevalent risk factors for thyroid cancer (TC), often appearing concurrently (31-33). Typically, small-sized, low-risk PTCs are indolent, boasting an excellent prognosis with survival rates approaching 99% to 100% over 5 years (34, 35). Active surveillance is increasingly recognized as a viable treatment strategy for patients diagnosed with minimally invasive papillary thyroid microcarcinoma (36, 37). Nevertheless, between 1% and 4% of PTC patients are found to have distant metastases at diagnosis, and 7% to 23% develop such metastases during subsequent follow-up (38, 39). Radioactive iodine therapy remains the primary treatment for metastatic PTC, although 60% of those with aggressive metastatic forms exhibit resistance to radioactive iodine therapy, leading to a diminished overall prognosis (39). The utilization of GLP-1RAs for managing diabetes and obesity has surged (40). Yet, the role of GLP-1RAs in the progression and management of TC is still debated (41, 42), limiting their use in patients with potentially malignant thyroid nodules. This study has elucidated the suppressive effects of the novel GLP-1RA, semaglutide, on TC cells through both in vitro and in vivo analyses, investigating its mechanisms via TAMs in curbing TC. These results offer crucial evidence supporting the reduction of both overdiagnosis and overtreatment of thyroid nodules. The potential of GLP-1RAs in the systemic treatment of TC, especially in cases resistant to iodine, remains to be confirmed through clinical trials assessing their safety and efficacy.

TAMs are a key immune cell population within the TME, playing dual roles in the progression of TC (43-45). M1 macrophages contribute to antitumor responses by recognizing and eliminating tumor cells through antigen presentation and phagocytosis (44). Conversely, M2 macrophages can promote tumor progression by enhancing angiogenesis and inducing immune tolerance (45). In this study, we found that while semaglutide significantly inhibited the progression of xenograft tumors in mice, it did not have a notable effect on PTC cell proliferation in vitro. This suggests that semaglutide's antitumor effects may be mediated through other cell types within the TME. Previous studies have demonstrated that certain GLP-1 analogs can modulate macrophage phenotypes in mice (46), influencing atherosclerosis. Therefore, we hypothesized that semaglutide might exert anticancer effects by regulating TAM phenotypes. Our analysis revealed a significant increase in CD86^+^ M1 macrophages and a decrease in CD206^+^ M2 macrophages in the semaglutide-treated group. Using an in vitro TAM model cocultured with PTC cells and THP-1 cells, we further showed that semaglutide inhibited the generation of M2-like TAMs while promoting M1-like TAMs. By inducing the polarization of THP-1 cells toward the M1 phenotype, semaglutide suppressed DNA replication and cell proliferation in PTC cells.

PPARG is a key member of the ligand-activated nuclear transcription factor superfamily and plays a crucial role in regulating a broad range of target genes (47, 48). Previous research has shown that PPARG inhibits the expression of target genes by preventing transcription factors and their cofactors from binding to specific promoter sites of inflammation-related genes, such as TNF-α, IL-1B, and NOS2 (49). Pharmacological activation of PPARG by natural or synthetic ligands has demonstrated significant anti-inflammatory effects (50). Additionally, PPARG is implicated in inducing immune cell apoptosis and inhibiting neutrophil function (51). In our study, semaglutide significantly inhibited PPARG expression in mouse PMs and THP-1 cells. Coadministration of a PPARG agonist partially reversed semaglutide-induced M1 polarization of THP-1 cells and promoted M2 polarization. These findings underscore the role of PPARG as a downstream regulator in semaglutide-induced reprogramming of TAM polarization.

Macrophage activation is closely tied to their metabolic state. M1 macrophages primarily generate energy through anaerobic glycolysis, while M2 macrophages maintain energy balance via oxidative glycolysis and fatty acid oxidation (52). In this study, we identified potential downstream target genes of PPARG related to glucose and lipid metabolism using bioinformatics analysis and qPCR, with a focus on *ACSL1* (Supplementary Fig. S4 (21), Fig. 5A-5E). *ACSL1*, a key enzyme in lipid metabolism, catalyzes the conversion of free fatty acids to fatty acyl-CoA, which participates in fatty acid oxidation, triglyceride synthesis, and phospholipid production (53). Dysfunction of *ACSL1* has been linked to conditions such as cardiac dysfunction (54), obesity (55), sepsis (56), and various cancers (57, 58). Research suggests that *ACSL1* plays a critical role in mediating the inflammatory phenotype of macrophages in diabetes and atherosclerosis (59, 60). In our study, semaglutide downregulated the transcription factor PPARG, thereby alleviating its suppression of the downstream molecule *ACSL1*, resulting in increased *ACSL1* expression (Fig. 5E). Knockdown of *ACSL1* in TAMs reduced lipid accumulation and promoted their polarization toward the M2 phenotype (Fig. 5F-5I). These findings suggest that semaglutide modulates TAM lipid metabolism and polarization through the PPARG/ACSL1 axis.

This study has several limitations. First, the absence of mouse thyroid cancer cell lines required the use of Balb/c nude mice for in vivo experiments with human PTC xenografts. The immune deficiencies in Balb/c nude mice could affect our understanding of semaglutide's mechanism. Second, due to this limitation, we used primary peritoneal macrophages from C57BL mice for our sequencing experiments. These cells were polarized in vitro with LPS and IL-4, which may not perfectly replicate the polarization state of TAMs in vivo. Additionally, while PTC, which originates from follicular epithelial cells, is the most common thyroid cancer, some malignancies arise from parafollicular cells (MTC). Due to the limited number of cases, our study focused primarily on PTC, potentially narrowing the applicability of our findings to other thyroid cancer types.

In summary, our study demonstrates that semaglutide does not directly influence the proliferation of thyroid cancer cells. Instead, it modulates macrophage lipid metabolism and polarization via the GLP-1R/PPARG/ACSL1 signaling pathway, leading to the induction of M1 polarization in TAMs and contributing to its antitumor effects. These findings highlight the potential of semaglutide to enhance therapeutic approaches, reduce unnecessary thyroid nodule screenings, and expand its clinical applications.

## Data Availability

The data sets produced through the current study are available from the corresponding author on reasonable request.

## Abbreviations

DEG: differentially expressed gene
FBS: fetal bovine serum
GLP-1: glucagon-like peptide 1
GLP-1RA: glucagon-like peptide 1 receptor agonist
IL-: interleukin
LPS: lipopolysaccharide
MTC: medullary thyroid carcinoma
PBS: phosphate-buffered saline
PCNA: proliferating cell nuclear antigen
PM: peritoneal macrophage
PTC: papillary thyroid carcinoma
qPCR: quantitative real-time polymerase chain reaction
TAM: tumor-associated macrophage
TC: thyroid cancer
TME: tumor microenvironment
